# Completely intracorporeal robotic-assisted laparoscopic ileovesicostomy: initial results

**DOI:** 10.1007/s11701-013-0443-y

**Published:** 2014-01-30

**Authors:** MaryEllen T. Dolat, Blake W. Moore, B. Mayer Grob, Adam P. Klausner, Lance J. Hampton

**Affiliations:** 1 Department of Surgery/Division of Urology, Virginia Commonwealth University School of Medicine, PO BOX 980118, Richmond, VA 23298-0118 USA; 2 Hunter Holmes McGuire Veteran’s Affairs Medical Center, Richmond, VA USA

**Keywords:** Urinary diversion, Neurogenic bladder, Robotics, Spinal cord injury

## Abstract

We present a series of robotic-assisted laparoscopic ileovesicostomies with bowel work performed completely intracorporeally. The four patients selected for this procedure were all diagnosed with neurogenic bladder and failed conservative medical therapy. Preoperative patient data included age, body mass index (BMI), and urodynamic (UD) study results. Intra-operative data included estimated blood loss (EBL), operative time, and intra-operative complications. Post-operative data included return to bowel function, post-operative complications, and length of hospital stay (LOS). All bowel work was completed intracorporeally with the exception of stoma maturation. Four robotic ileovesicostomies were performed. Pre-operative urodynamic study results showed either elevated detrusor pressures or limited bladder capacities in addition to the inability to perform self-catheterization. The mean patient age was 40 years and mean BMI was 26 kg/m^2^. Average EBL and operative time were 131 ml and 290 min, respectively. No intra-operative complications occurred. Bowel function, as defined as flatus, returned on average 3.8 days after surgery and average LOS, defined as discharge home or discharge to the spinal cord unit, was 7.5 days. Mean follow-up time was 25.8 months. Post-operative urodynamic studies revealed low stomal leak point pressure (<10 cmH_2_O). This study is the first to describe a completely intracorporeally robotic-assisted laparoscopic ileovesicostomy with safe and effective outcomes after more than 2 years of follow-up.

## Introduction

Many patients with neurogenic bladders require treatment to reduce elevated bladder pressure and prevent subsequent renal damage. These individuals are often managed with conservative therapies such as intermittent catheterization or, less ideally, long-term indwelling catheters. Ileovesicostomy is a long-term surgical management option for patients who have failed medical or other conservative therapies [1–6]. Many studies have shown a reduction in chronic urinary tract infections and an improvement in quality of life in carefully selected patients who undergo this procedure [1–6].

Traditionally, ileovesicostomy has been performed using an open technique that is associated with post-operative complications including wound infection, urethral incontinence, and extended length of hospital stay (LOS) [7]. A minimally invasive technique was first described for laparoscopic enterocystoplasty and ileovesicostomy by Elliott et al. [9] and Abrahams et al. [10] but there is sparse literature describing an intracorporeal laparoscopic bowel-to-bowel anastomosis [8, 11]. In 2009, Vanni and Stoffel [12, 13] published initial results of robotic-assisted ileovesicostomies; however, the bowel work was performed extracorporeally. The goal of this retrospective study is to report initial results including pre-operative, intra-operative, and post-operative variables for robotic-assisted ileovesicostomy with a focus on a completely intracorporeal bowel work and the potential benefits compared to extracorporeal robotic-assisted ileovesicostomy.

## Materials and methods

This study was approved by the Institutional Review Boards at the Virginia Commonwealth University School of Medicine and the Hunter Holmes McGuire Veterans Affairs Medical Center. Patients who underwent robotic-assisted laparoscopic ileovesicostomy between April 2010 and April 2011 were retrospectively identified and reviewed. The four patients selected for this procedure were all diagnosed with neurogenic bladder and failed conservative medical therapy. All patients were unwilling or unable to perform intermittent catheterization and chose ileovesicostomy after all available options were presented. Pre-operative patient data included age, body mass index (BMI), and urodynamic (UD) study results. Intra-operative data included estimated blood loss (EBL), operative time, and intra-operative complications. Post-operative data included return to bowel function, post-operative complications, and LOS. All bowel work was completed intracorporeally with the exception of stoma maturation. The same surgical team, which included a robotic fellowship trained surgeon and a surgeon with prior open ileovesicostomy experience, performed all surgeries. All data are presented as mean ± standard deviation (SD).

### Surgical technique

**Fig. 1:**
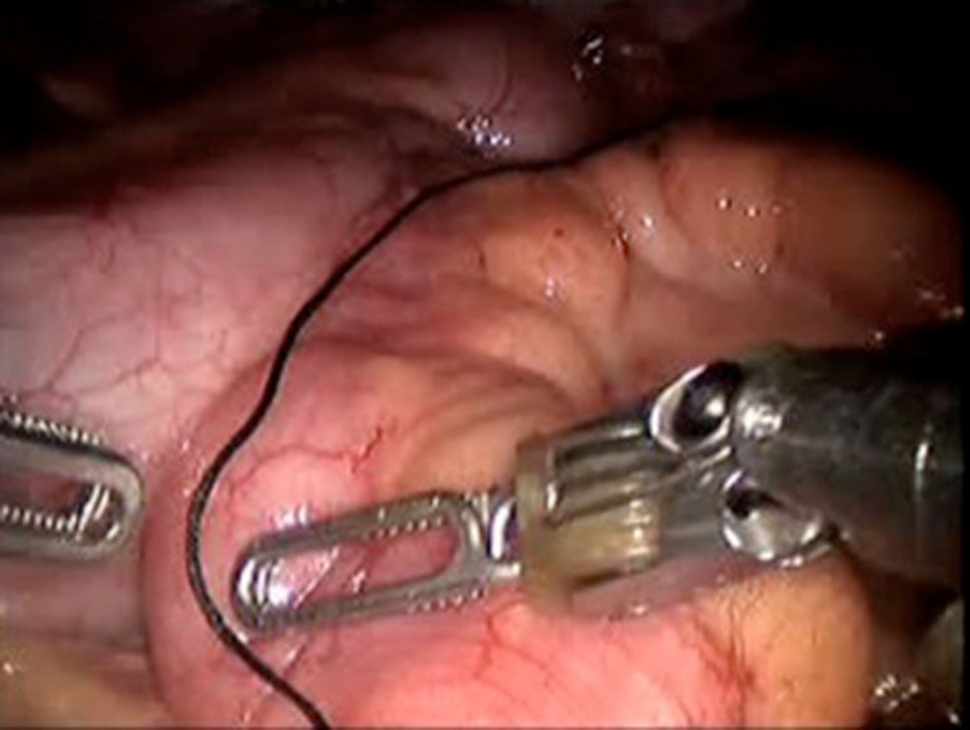
Identification and measurement of ileum

**Fig. 2:**
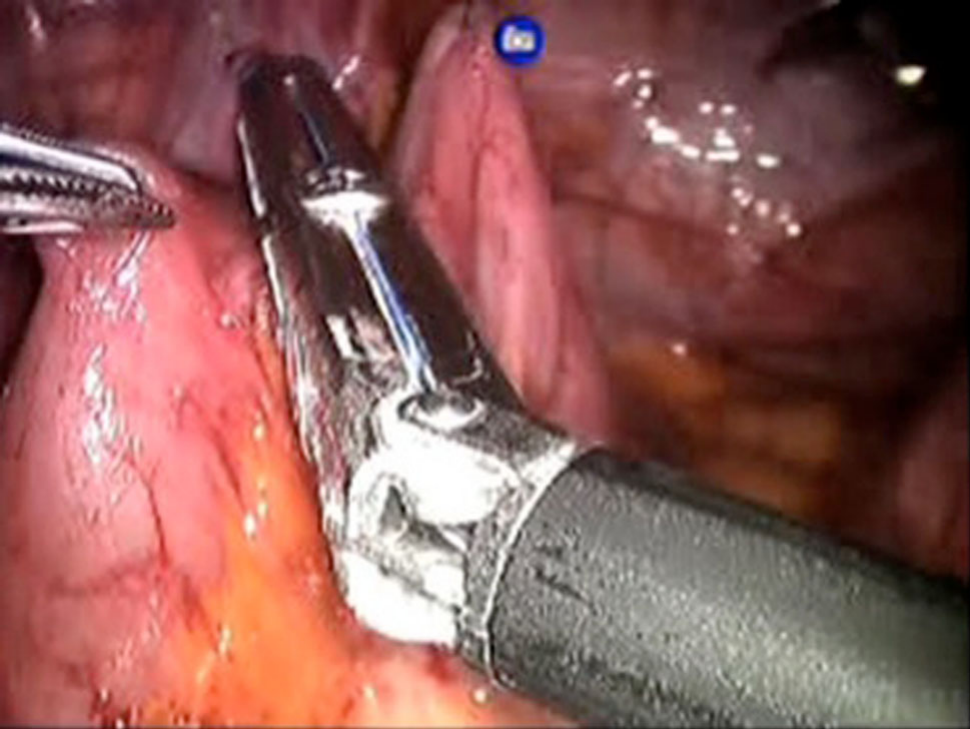
Intracorporeal bowel manipulation

**Fig. 3:**
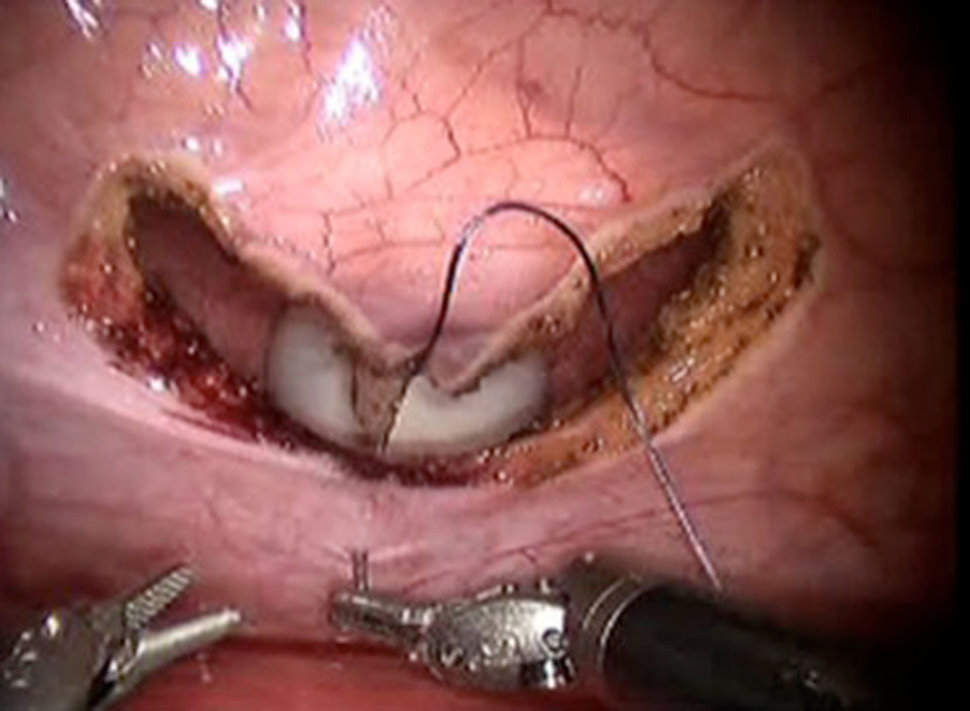
Start of ileal–vesical anastomosis

The da Vinci Surgical System robot (Intuitive Surgical, Sunnyvale, CA, USA) was used for the entire procedure and a video example of the entire surgical procedure for one of the patients included is available online at http://www.youtube.com/watch?v=QLBzoUEWVIg. The patients were placed in dorsal lithotomy position in steep Trendelenburg for the operation. Three robotic ports and two assistant ports were used in positions similar to that of a robotic-assisted laparoscopic prostatectomy. A 3-0 silk holding stitch was placed in the ilium 15 cm proximal to the ileocecal valve and a separate 3-0 silk suture was placed 15 cm proximal to the first suture (Figs. 1, 2). A surgical stapler was then used to isolate a 15-cm loop of ileum and perform a functional end-to-end anastomosis. Next, a U-shaped bladder flap was created using electrocautery. Then, after spatulation of the antimesenteric side, the proximal end of the ileal loop was sutured to the bladder (Fig. 3). The bladder was irrigated to ensure no leakage. A suture was then placed on the distal end of the ileal loop and brought through one of the ports or through the skin with a Carter–Thomason device (Cooper Surgical, Trumball, CT, USA) depending on the desired location of the stoma. A 15-French round Jackson–Pratt drain was placed prior to undocking. The stoma was then created.

## Results

Four robotic-assisted ileovesicostomies were performed between April 2010 and April 2011. The mean patient age was 40 years (SD 14.9, range 29–60 years) and mean BMI was 26 kg/m^2^ (SD 2.8, range 20–26.2 kg/m^2^). All patients had neurogenic bladder dysfunction: either high-pressure detrusor overactivity that was refractory to medical therapy or inability or unwillingness to perform intermittent catheterization. Pre-operative urodynamic study results showed either elevated detrusor pressures or limited bladder capacities in addition to the inability to perform self-catheterization. Average EBL and operative time were 131 ml (SD 80, range 75–250 ml) and 289.5 min (SD 35.7, range 244–330 min), respectively. No intra-operative complications occurred. Bowel function, as defined as flatus, returned on average 3.8 days (SD 1.0, range 3–5 days) after surgery and average LOS, defined as discharge home or discharge to the spinal cord unit, was 7.5 days (SD 2.6, range 5–11 days). Mean follow-up time was 25.8 months (SD 9.7, range 16–39 months). Table 1 shows our initial results side-by-side with a case series of robotic-assisted laparoscopic ileovesicostomies with extracorporeal bowel work published by Vanni et al. from the Lahey clinic [13].

**Table 1:** Robotic-assisted ileovesicostomy: comparison of initial results of this study with Lahey clinic initial results

	This study initial results	Lahey clinic initial results
Preoperative data		
No. of patients	4	9
Mean age (years)	45 ± 14.9	53 ± 11.1
Mean BMI (kg/m^2^)	25.9 ± 2.8	29.0 ± 6.1
Intra-operative data		
EBL (ml)	131 ± 80.0	100 ± 71.9
Total operative time (min)	289.5 ± 35.7	330 ± 72
Conversion to open	0	1
Postoperative data		
Length of hospital stay (days)	7.5 ± 2.6	7.7 ± 1.3
Return of bowel function (days)	3.3 ± 1.0	4.8 ± 1.3
No. of major complications	0	0
Mean follow-up time (months)	25.8 ± 9.7	14 ± 7.4

*EBL* estimated blood loss, *BMI* body mass index

In the peri-operative period (<30 days from time of surgery), there was one Clavien–Dindo grade I complication in a female patient who developed a urinary tract infection requiring antibiotics on post-operative day 6. This patient, with pre-existing urethral erosion from a chronic indwelling catheter, had continued urethral incontinence requiring peri-urethral bulking injection 7 months post-operatively and subsequent sub-urethral sling 10 months post-operatively with resolution of symptoms. All patients were offered a post-operative urodynamic study at least 6 months after surgery (one patient declined). Three of the four patients had post-operative urodynamic studies revealing an average stomal leak point pressure of 5.3 cmH_2_O (SD 4.5, range 1–10 cmH_2_O) and average volume at which leakage occurred of 141.3 ml (SD 138.3, range 46–300 ml). One patient had a low stomal leak point pressure, but had elevated residual volume (approximately 300 ml). Cystoscopic evaluation demonstrated small caliber vesicostomy. Although counseled that a low pressure system provided a functionally acceptable result, the patient remained concerned about potential infection from an elevated residual volume and an open conversion of ileovesicostomy to ileal conduit was performed approximately 16 months after his initial surgery.

## Discussion

Ileovesicostomy is an effective long-term bladder management for patients with neurogenic bladder who are unable or unwilling to perform intermittent catheterizations [1–6]. This case series is the first to our knowledge to report a completely intracorporeal robotic-assisted ileovesicostomy procedure. There have been several studies in the literature highlighting the advantages of minimally invasive surgery in urology [14, 15]. Specifically, the literature reports decreased intra-operative blood loss, reduced hospital stay, and less post-operative pain [16]. In recent years minimally invasive surgery has been adapted to the ileovesicostomy.

In 2009, Vanni and Stoffel [12, 13] reported initial results of a case series of nine robotic-assisted ileovesicostomy procedures. Table 1 compares our initial results to their first published robotic-assisted ileovesicostomy data. This side-by-side comparison suggests similar results for EBL, total operative time, and LOS. The data from the Lahey clinic initial robotic-assisted ileovesicostomy was also compared to data from open ileovesicostomies performed at the same institution. Additionally, our initial results are similar to other open series [2, 17].

The fundamental difference between our case series and those reported in the literature lies in surgical technique. Our surgical technique allows for intracorporeal laparoscopic bowel work, whereas previously published case series describe an extracorporeal technique in which the divided bowel is pulled through an incision and an extracorporeal stapled end-to-end anastomosis is performed. The benefits of an extracorporeal bowel-to-bowel anastomosis have been discussed in the literature [9, 18]. However, a study by Abhrams et al. [9] favors a completely intracorporeal anastomosis, suggesting that it decreases unnecessary tension on the bowel and may prevent mesenteric thrombosis/ischemia. Additionally, the minimally invasive technique decreases bowel manipulation which may shorten the time to return of bowel function and decrease the incidence of post-operative ileus [9]. Completely intracorporeal bowel work also results in a smaller incision, possibly decreasing pneumoperitoneal leak and decreasing the risk of incisional hernia [9].

Our case series, in conjunction with other minimally invasive outcomes, shows that a minimally invasive ileovesicostomy procedure may decrease the risk of wound infection [19, 20]. Additionally, after reviewing preliminary data of open vs. robotic-assisted laparoscopic ileovesicostomy at our institution (data not shown), post-operative return of bowel function was improved in those patients treated robotically. A larger cohort of patients is needed to make more definitive conclusions about the long-term potential benefits of robotic-assisted ileovesicostomy.

## Conclusion

The results of this study show that the first four cases of completely intracorporeal robotic-assisted ileovesicostomy were successfully completed without open conversion or major complications. Long-term studies are needed to examine the benefits of a minimally invasive approach and, specifically, the possible benefits of intracorporeal bowel work.

## References

[1] Atan A, Konety BR, Nangia A, Chancellor MB (1999). Advantages and risks of ileovesicostomy for the management of neuropathic bladder. Urology.

[2] Gauthier A, Winters JC (2003). Incontinent ileovesicostomy in the management of neurogenic bladder dysfunction. Neurourol Urodyn.

[3] Leng WW, Faerber G, Del Terzo M, McGuire EJ (1999). Long-term outcome of incontinent ileovesicostomy management of severe lower urinary tract dysfunction. J Urol.

[4] Mutchnik SE, Hinson JL, Nickell KG, Boone TB (1997). Ileovesicostomy as an alternative form of bladder management in tetraplegic patients. Urology.

[5] Zimmerman WB, Santucci R (2009) Ileovesicostomy update: changes for the 21st century. Adv Urol 801038. doi 10.1155/2009/801038 10.1155/2009/801038 PMC2768112 19884984

[6] Hellenthal N, Short S, O’Connor RC, Eandi J, Yap S, Stone A (2009). Incontinent ileovesicostomy: long-term outcomes and complications. Neurourol Urodyn.

[7] Tan H, Stoffel J, Daignault S, McGuire E, Latini J (2008). Ileovesicostomy for adults with neurogenic bladders: complications and potential risk factors for adverse outcomes. Neurourol Urodyn.

[8] Hsu THS, Rackley R, Abdelmalak J, Tchetgen M, Madjar S, Vasavada S (2002). Laparoscopic ileovesicostomy. J Urol.

[9] Abrahams H, Rahman N, Meng M, Stoller M (2003). Pure laparoscopic ileovesicostomy. J Urol.

[10] Elliott S, Meng M, Anwar H, Stoller M (2002). Complete laparoscopic ileal cystoplasty. Urology.

[11] Yohannes P, Khan A, Francis K, Sudan R (2004). Robot-assisted bricker ileoureteral anastomosis during intracorporeal laparoscopic ileal conduit urinary diversion for prostatocutaneous fistula: case report. J Endourol.

[12] Vanni A, Stoffel J (2011). Ileovesicostomy for the neurogenic bladder patient: outcome and cost comparison of open and robotic assisted techniques. Urology.

[13] Vanni A, Cohen M, Stoffel J (2009). Robotic-assisted ileovesicostomy: initial results. Urology.

[14] Gill IS, Kerbe K, Meraney AM, et al. (2002) Basics of laparoscopic urologic surgery. In: Walsh PC, Retik AB, Vaughan DE, et al (eds) Campbell’s urology, vol 4. Philadelphia, WB Saunders, pp 3455–3505

[15] Balaji KC, Yohannes P, McBride C, Oleynikov D, Hemstreet G (2004). Feasibility of robot-assisted totally intracorporeal laparoscopic ileal conduit urinary diversion: initial results of a single institutional pilot study. Urology.

[16] Smith J, Herrell SD (2005). Robotic-assisted laparoscopic prostatectomy: do minimally invasive approaches offer significant advantages?. J Clin Oncol.

[17] Passerotti C, Nguyen H, Lais A (2008). Robot-assisted laparoscopic ileal bladder augmentation: defining techniques and potential pitfalls. J Endourol.

[18] Gudziak MR, Tiguert R, Puri K, Gheiler EL, Triest JA (1999). Management of neurogenic bladder dysfunction with incontinent ileovesicostomy. Urology.

[19] Romy S, Eisenring MC, Bettschart V, Petignat C, Francioli P, Troillet N (2008). Laparoscope use and surgical site infections in digestive surgery. Ann Surg.

[20] Khan MN, Fayyad T, Cecil TD (2007). Laparoscopic versus open appendectomy: the risk of postoperative infectious complications. JSLS..

